# Minor impact of probiotic bacteria and egg white on *Tenebrio molitor* growth, microbial composition, and pathogen infection

**DOI:** 10.3389/finsc.2024.1334526

**Published:** 2024-03-01

**Authors:** Carlotta Savio, Pascal Herren, Agnès Rejasse, Alfredo Rios, William Bourelle, Annette Bruun-Jensen, Antoine Lecocq, Joop J. A. van Loon, Christina Nielsen-LeRoux

**Affiliations:** ^1^ University of Paris Saclay, INRAE, Micalis, Jouy-en-Josas, France; ^2^ Laboratory of Entomology, Department of Plant Sciences, Wageningen University, Wageningen, Netherlands; ^3^ Department of Plant and Environmental Sciences, University of Copenhagen, Frederiksberg, Denmark; ^4^ UK Centre for Ecology & Hydrology, Crowmarsh Gifford, Wallingford, United Kingdom; ^5^ Faculty of Biological Sciences, University of Leeds, Leeds, United Kingdom; ^6^ Ynsect, Evry, France

**Keywords:** yellow mealworm, entomopathogen, probiotics, insect health, *Bacillus thuringiensis*, *Pediococcus pentosaceus*, *Lactiplantibacillus plantarum*

## Abstract

The industrial rearing of the yellow mealworm (*Tenebrio molitor*) for feed and food purposes on agricultural by-products may expose larvae and adults to entomopathogens used as biocontrol agents in crop production. Bacterial spores/toxins or fungal conidia from species such as *Bacillus thuringiensis* or *Metarhizium brunneum* could affect the survival and growth of insects. Therefore, the aim of this study was to investigate the potential benefits of a wheat bran diet supplemented with probiotic bacteria and dried egg white on larval development and survival and its effects on the gut microbiome composition. Two probiotic bacterial species, *Pediococcus pentosaceus* KVL B19-01 and *Lactiplantibacillus plantarum* WJB, were added to wheat bran feed with and without dried egg white, as an additional protein source, directly from neonate larval hatching until reaching a body mass of 20 mg. Subsequently, larvae from the various diets were exposed for 72 h to *B. thuringiensis*, *M. brunneum*, or their combination. Larval survival and growth were recorded for 14 days, and the bacterial microbiota composition was analyzed using 16S rDNA sequencing prior to pathogen exposure and on days 3 and 11 after inoculation with the pathogens. The results showed increased survival for *T. molitor* larvae reared on feed supplemented with *P. pentosaceus* in the case of co-infection. Larval growth was also impacted in the co-infection treatment. No significant impact of egg white or of *P. pentosaceus* on larval growth was recorded, while the addition of *Lb. plantarum* resulted in a minor increase in individual mass gain compared with infected larvae without the latter probiotic. On day 14, *B. thuringiensis* was no longer detected and the overall bacterial community composition of the larvae was similar in all treatments. On the other hand, the relative operational taxonomic unit (OTU) abundance was dependent on day, diet, and probiotic. Interestingly, *P. pentosaceus* was present throughout the experiments, while *Lb. plantarum* was not found at a detectable level, although its transient presence slightly improved larval performance. Overall, this study confirms the potential benefits of some probiotics during the development of *T. molitor* while underlining the complexity of the relationship between the host and its microbiome.

## 1 Introduction

The predicted increased demand for protein is a driver to exploit several protein sources to preserve human health while mitigating the environmental impact of protein production (1–3). Among the explored options, some insect species have been promoted by the Food and Agricultural Organization of the United Nations (4) as a promising protein source due to their nutritional value (5) and their suitability for the mass rearing of some species (6). Among more than 2,000 edible insect species (7), the yellow mealworm (*Tenebrio molitor* L.) (Coleoptera: Tenebrionidae) is one of those authorized for feed and food production (8). The larval life stage has between 11 and 19 instars (9) depending on the environmental and nutritional conditions. These parameters can also influence the chemical composition of the insect, leading to research to improve the rearing conditions and diet composition to optimize protein production (10).

In mass-rearing environments, insects may be exposed to entomopathogens that can negatively affect their performance (11). The source of these pathogens can be found in organic cereal products used as feed for insects, as these products possibly contain biocontrol agents used during crop production or naturally present in the environment, such as the entomopathogens *Bacillus thuringiensis* (12) and *Metarhizium brunneum* (13).

While good manufacturing practice (GMP) and Hazard Analysis and Critical Control Point (HACCP) have been proposed for reducing the rate of infection and avoiding pathogen transmission (14, 15), several studies have also highlighted the important role of symbionts in maintaining host health (16–18). Interactions between beneficial symbionts and insects from various orders have already been proven to be effective in reducing pathogen incidence. For example, the honeybee (*Apis mellifera* L.) can benefit from hive supplementation of *Lactobacillus* spp. against infections by *Paenibacillus larvae*, the causal agent of the American foulbrood disease. In this case, the reduced mortality and pathogen loads suggest a higher protection against pathogens through primed immunity from *Lactobacillus* (19). The effects of prokaryotic probiotics have also been studied in insects reared for sterile insect technique programs, such as the medfly (*Ceratis capitata* L.) (20, 21), and in insects reared for food and feed, such as the black soldier fly (*Hermetia illucens* L.) (22–24) and *T. molitor* (25, 26).

Nonetheless, the role of the microbiota of *T. molitor* in maintaining health and the effects of live and deactivated potential probiotic strains on larval performance while exposed to pathogens are yet to be investigated. In this study, *T. molitor* larvae were reared on two diets: a plain wheat bran (WB) diet and a WB diet enriched in protein (in the form of dried egg white, WE). Each diet was supplemented with two potential probiotic bacterial species—*Pediococcus pentosaceus* KVL B19-01 (25) and *Lactiplantibacillus plantarum* WJB (27)—in their live and deactivated forms to quantify the larval performance and microbial community composition in rearing conditions mimicking the environment of mass-rearing systems.

The choice of the probiotic strains was based on their origin (18, 25), their previously examined efficacy on *T. molitor* larvae in laboratory rearing conditions (25), and on malnourished *Drosophila melanogaster* (27), which showed increased performance in both cases. Furthermore, we analyzed whether the presence of these probiotics could decrease the impact of two entomopathogens: the bacterium *B. thuringiensis* sv. *morrisoni* biovar *tenebrionis* (Btt) and the fungus *M. brunneum*. The composition of the bacterial gut microbiota was also monitored to assess the persistence of the probiotics and Btt in the larvae and its possible modulation due to the pathogens, diet, and probiotics.

## 2 Materials and methods

### 2.1 Preparation of probiotics and postbiotics

The *P. pentosaceus* isolate KVL B19-01 was obtained from the University of Copenhagen (Department of Plant and Environmental Sciences, Denmark) and *Lb. plantarum* WJB obtained from François Leulier (Ecole Normale Supérieure, Lyon, France) (27), which were cultivated in de Man, Rogosa, and Sharpe (MRS) broth (28) for 24 h at 30°C in anaerobic (oxygen <20%, closed jars with AnaeroGen 2.5-L bag; Oxoid, Thermo Fisher, Paris, France) and aerobic conditions. After washing the cultures, freeze drying was performed at −85°C and 0.01 mbar for 48 h (Alpha 2-4 LSCbasic, Martin Christ, Osterode am Harz, Germany). The freeze-dried bacteria were stored at −70°C. Postbiotic cells (deactivated cells) were obtained by heat treatment of the freeze-dried cultures at 121°C for 15 min in an autoclave. The efficiency of the freeze drying and the deactivation processes was verified using classical microbiological methods by enumerating the colony-forming units (CFUs) of both strains on MRS agar after 24 h of incubation at 30°C.

### 2.2 Experimental diet preparation

The control diets for *T. molitor* larvae were composed of WB or WE (Louis Francois, Croissy Beaubourg, France). The bacteria *P. pentosaceus* and *Lb. plantarum* were added to the control diets to obtain a load of 10^9^ CFU/mg diet (50 mg diet and 0.5 mg freeze-dried bacteria). The same amount of deactivated freeze-dried bacteria was added to the two control diets following the protocol described by Lecocq et al. (25).

### 2.3 *Tenebrio molitor* rearing and probiotic provision

**Figure 1:**
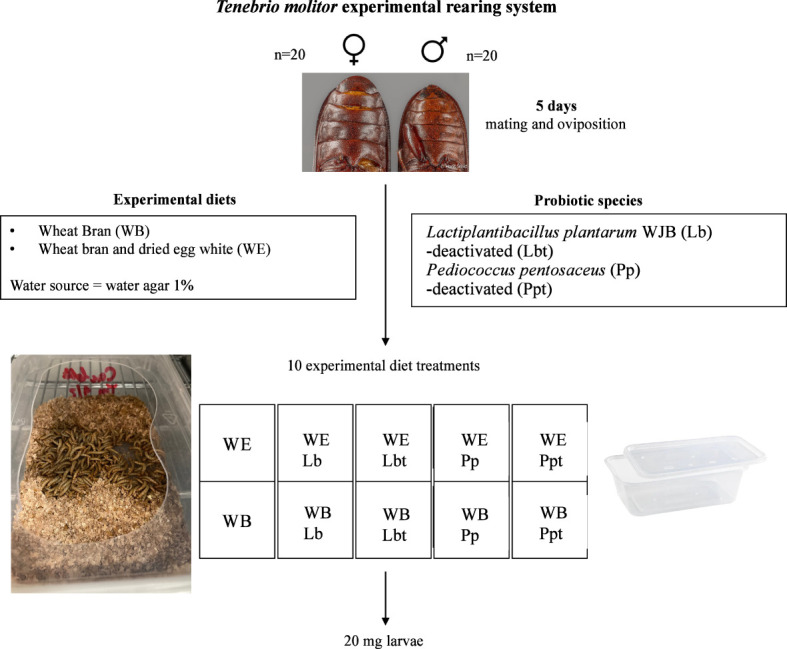
*Tenebrio molitor* experimental rearing system. Adults mate on a wheat bran (*WB*) diet or dried egg white and wheat bran (*WE*) diet supplemented with the selected probiotic species *Lactiplantibacillus plantarum* WJB (*Lb*) or *Pediococcus pentosaceus* KVL B19-01 (*Pp*) freeze dried in their live or deactivated (indicated by “*t*”) form.

*T. molitor* larvae were sourced from the private company YNSECT (Evry, France). Adult beetles (20 males and 20 females, >7 days after imago eclosion) were added into 750-ml plastic boxes (15 cm × 9.5 cm = 142.5 cm^2^; Stackables, UK) containing 50 g of one of the 10 experimental diets (see **Table 1**). After 5 days, the adults were removed and the hatching larvae received 10 g water agar (1%, *w*/*v*) twice a week starting 1 week after removal of the adults. The pathogen infection experiments started when the individuals reached a body mass of 20 mg (**Figure 1**). Individual larval weight was recorded gravimetrically, and only 20 mg larvae were selected for the assay (1-mg precision; Sartorius, BP211D, Göttingen, Germany). Rearing took place at 28°C in complete darkness. Water containers were added at the bottom of the incubator to reach a relative humidity (RH) of approximately 65%.

**Table 1:** Composition of the experimental diets provided to *Tenebrio molitor* larvae during the assay.

Experimental diet										
	WB	WB_Pp	WB_Ppt	WB_Lb	WB_Lbt	WE	WE_Pp	WE_Ppt	WE_Lb	WE_Lbt
Ingredient (%)										
Wheat bran (WB)	100	100	100	100	100	90	90	90	90	90
Dried egg white (WE)						10	10	10	10	10
*Pediococcus pentosaceus* KVL B19-01 (Pp)		X					X			
*P. pentosaceus* KVL B19-01 deactivated (Ppt)			X					X		
*Lactiplantibacillus plantarum* WJB (Lb)				X					X	
*Lb. plantarum* WBJ deactivated (Lbt)					X					X

### 2.4 Culture of bacteria and fungi and preparation of suspension for pathogen infection

#### 2.4.1 Bacillus thuringiensis

*B. thuringiensis* sv. *morrisoni* biovar *tenebrionis* isolate 4AA1 (Btt) (*Bacillus* Genetic Stock Center, Ohio State University, Columbus, OH, USA) was grown in HCT medium (5 g tryptone, 2 g Bacto casamino acids, 6.8 g KH_2_PO_4_, 0.1 g MgSO_4_, 0.002 g MnSO_4_, 0.014 g ZnSO_4_, 0.15 g CaCl_2_, and 0.022 g ammonium ferric citrate in 1 L dH_2_O) (29) for 96 h at 30°C, 200 rpm. The bacterial cultures were then washed twice with 20 ml phosphate-buffered saline (PBS), pH 7.4, with a centrifugation step at 15,000 rpm at 4°C and a final dilution in 20 ml PBS. The presence of spores and toxin crystals was verified by light microscopy observation. Bradford protein analysis (30) using the Bio-Rad microprotein assay was performed to quantify the protein content of the Btt toxin crystal, and 10% SDS-PAGE was used to qualify their nature (relative part of the Cry3Aa 65-kDa toxin). The results were used to calculate the toxin content (in micrograms per microliter) of the Btt culture.

The infection suspension was exposed to high-temperature treatment (80°C) for 10 min to deactivate vegetative cells in order to provide the insects with Btt spores only. The spore load was then determined by CFU counting on Luria–Bertani agar (LBA) after an incubation period of 24 h at 30°C. The ratio of the toxin/spore suspension in the feed during the assay was expressed in micrograms toxin per milligram feed, and the volume of suspension added in the feed was 100 µl/100 mg feed. The concentration of Btt toxin used for the infection was prepared by concentrating the infection suspension in order to obtain a final load of 2.5 μg toxin/mg feed and 2 × 10^7^ spores CFU/mg feed.

#### 2.4.2 Metarhizium brunneum

The *M. brunneum* isolate KVL 12-30 (Department of Plant and Environmental Sciences, University of Copenhagen) was grown on Petri dishes (9 cm diameter, triple vented) containing Sabouraud dextrose agar (SDA; 65 g/L) at 23°C in complete darkness for 21 days. The conidia were harvested with a Drigalski spatula by pouring 10 ml of Tween 20 (0.05%) into the Petri dishes. Two washing steps with 15 ml of Tween 20 (0.05%) with centrifugation (3,000 rpm for 3 min) to discard the supernatant each time were followed by a dilution step with water to obtain a 1,000 times diluted suspension. The conidia were counted under a light microscope at ×400 magnification after pouring 20 μl of the suspension into a 0.2-mm Fuchs-Rosenthal hemocytometer. The conidia were counted in four squares (one square containing 16 cells) diagonally starting on the lower left corner. The concentration (conidia per milliliter) was then calculated by multiplying the average number of conidia per square with 5,000,000/ml (1,000 times dilution/0.0002 ml volume). The spore suspensions were then prepared from the stock suspension using the following formula: *C*_1_ * *V*_1_ = *C*_2_ * *V*_2_ (where *C* is the concentration and *V* is the volume). All spore suspensions were prepared and used for inoculation on the same day. A germination test was performed to ensure the ability of the fungal conidia to germinate. On the day of the assay, 100 μl of the infection suspension was plated on the SDA medium and incubated for 24 h at 28°C at 65% RH in the dark. Conidial germination was recorded by calculating the percentage of conidia per Petri dish in 3 Petri dishes. Values of conidial germination above 99% were accepted, which indicate that the fungi were able to germinate in the conditions of infection. The *M. brunneum* conidial suspension used for the infection was 30,000 conidia/mg diet.

### 2.5 *Tenebrio molitor* co-infection assay

*T. molitor* larvae were infected with *B. thuringiensis* sv. *morrisoni* biovar *tenebrionis* 4AA1 (Btt) and *M. brunneum* KVL 12-30. The pathogens were provided in the diet singly or in combination to investigate the effects of co-infection on *T. molitor* larval performance and on the larval microbial community composition.

#### 2.5.1 Pathogen inoculation of T. molitor and subsequent actions

**Figure 2:**
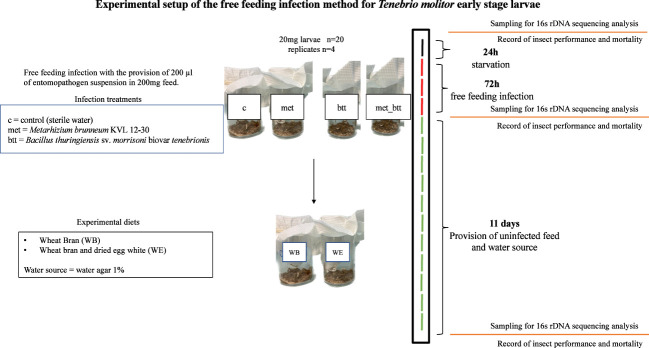
Experimental setup of the inoculation of *Tenebrio molitor* larvae with the entomopathogens *Bacillus thuringiensis* sv. *morrisoni* biovar *tenebrionis* (*btt*) and *Metarhizium brunneum* KVL 12-30 (*met*) or with both pathogens (*met_btt*) and sampling for microbiota analysis. After 24 h of starvation (*black line*), the larvae were exposed to the entomopathogens or sterile water (*c*) with the free feeding method for 72 h (*red lines*), followed by 11 days of provision of uninfected feed [wheat bran with egg white (*WE*) or wheat bran (*WB*) diet] and water agar every 2 days (*green lines*).

Larvae of *T. molitor* with a body mass of 20 mg from the 10 different treatments were selected for the infection experiments. Groups of 20 larvae were first placed without feed in sterile plastic cups (60 ml; Qualibact^®^ resistant 95 kPa, Labelians, France) to obtain a density of 0.07 g/cm^2^ for 24 h (**Figure 2**) in four replicates for each infection treatment. An amount of 100 mg of diet, composed of a mixture of WB (90 mg) and WE (10 mg) or WB alone, was poured into each cup. A volume of 100 µl of the Btt suspension or the *M. brunneum* suspension or 50 µl Btt + 50 µl *M. brunneum* suspension or 100 µl sterile water as the negative control treatment, giving 1 μl/mg feed, was poured homogenously in the feed for inoculation. A wet paper was inserted into the cups to maintain the humidity. Subsequently, the cups were covered with tissue paper and incubated at 28°C for 72 h. After 72 h, the dead insects, frass, and remaining feed were mechanically separated by sieving (Ø = 200 mm; pore sizes, 2 mm, 1.4 mm, and 500 μm) (Retsch, Eragny sur Oise, France). The larvae (about 400 mg larval biomass) were then transferred into four new sterile cups per infection treatment containing the WB diet (30% of the larval biomass) and 1% water agar (about 60% of the larval biomass). The number of surviving larvae and the weight of all larvae in the cup were subsequently recorded every 2 days for 11 days (**Figure 2**).

#### 2.5.2 Statistical analysis

Statistical analysis of the survival, relative growth, and the individual mean mass (IMM) of the larvae was performed using R 4.2.2 (31) on three replicates. Each of these variables was modeled as a function of the probiotic, diet, pathogen, and their interactions.

A generalized linear model with a binomial error distribution was used to model the number of surviving larvae. Given that one of the categories presented complete separation (i.e., all larvae survived), a bias reduction method (brglmFit) from the R package brglm2 (32) was used. Wald tests were then used to assess the statistical significance of the factor effects using the lmtest package. Thereafter, estimated marginal means and contrasts (emmeans package) (33) were used to compare differences between the different treatments and the control category (no pathogen and no probiotics) within a given diet and pathogen inoculation category. The *p*-values were adjusted using an approximation of the Dunnett’s method. Similarly, contrasts were used to estimate the differences in survival between diets with the same probiotic and pathogen status.

The IMM was calculated by dividing the biomass of the alive larvae at the end of the assay by the total number of larvae, and relative growth was determined by dividing the difference between the final and the initial weight of the population (all larvae per cup) by the initial weight following the methods previously used by Dupriez et al. (34). Analysis of variance (ANOVA) was used to test the statistical significance of the factors previously mentioned on larval relative growth and IMM. Shapiro-Wilk’s and Bartlett’s tests were run on the residuals of the ANOVA model to test for normality and homogeneity of the variances among factor groups. No departure from these conditions was found (*α* = 0.05). Thereafter, estimated marginal means and contrasts were used for survival. The *p*-values for contrasts among factors were adjusted with Tukey’s and Dunett’s methods for relative growth and IMM, respectively.

### 2.6 Microbiota composition

Sequencing of 16S rDNA was performed to explore the ability of the probiotics to persist in the gut of *T. molitor* and their effects on the composition of larval microbiota in the presence and absence of pathogen infection and the impact of the diet.

#### 2.6.1 DNA extraction

Larvae were sampled from each treatment when an individual body mass of 20 mg was reached (prior to pathogen exposure, day 0), 72 h after pathogen infection treatments (day 3), and 11 days after pathogen removal (day 14). DNA extraction was performed on 4 replicates composed of 3 individuals each (*n* = 12 larva per treatment per day) (**Figure 2**). The larvae were surface sterilized with 3 washing steps consisting of successive immersions in diethyl pyrocarbonate (DEPC) water, EtOH 70%, and DEPC water for 30 s. Three larvae were pooled and then ground with liquid nitrogen. DNA extraction was performed with the DNeasy® PowerSoil® Pro Kit (Qiagen®, Hilden, Germany). The quality of the genetic material was determined by NanoDrop and Qubit analysis. Samples were stored at −80°C.

#### 2.6.2 16S rDNA sequencing analysis

The V3–V4 hypervariable regions of the ribosomal RNA subunit 16S rDNA gene were amplified from the DNA extracts during the first PCR step with the universal primers PCR1F_343 and PCR1_R784 (**Table 2**), which are fusion primers as described by Nadkarni et al. (35). This PCR was performed using 2 U of a DNA-free Taq DNA polymerase and 1× Taq DNA polymerase buffer (MTP Taq DNA Polymerase; Sigma-Aldrich, St. Louis, MO, USA). The buffer was completed with 10 nmol of a dNTP mixture (Sigma-Aldrich, St. Louis, MO, USA), 15 nmol of each primer (Eurofins, Luxembourg), and nuclease-free water (Qiagen, Hilden, Germany) in a final volume of 50 μl.

**Table 2:** List of primers used for the PCR1 and PCR2 steps of the 16S rDNA sequencing analysis.

Primer name	Primer sequence (5′–3′)	rRNA operon binding site
PCR1F_343	CTTTCCCTACACGACGCTCTTCCGATCT-ACGGRAGGCAGCAG partial P5 adapter–primer	V3–V4
PCR1_R784	GGAGTTCAGACGTGTGCTCTTCCGATCTTACCAGGGTATCTAATCCT partial P7 adapter–primer	V3–V4
PCR2_P5F	AATGATACGGCGACCACCGAGATCTACACT-CTTTCCCTACACGAC partial P5 adapter–primer targeting primer 1F	
PCR2_P7R	CAAGCAGAAGACGGCATACGAGAT-NNNNNN-GTGACT-GGAGTTCAGACGTGT partial P7 adapter including index–primer targeting primer 1R	

The PCR reaction was carried out in a T100 Thermal cycler (Bio-Rad, Hercules, CA, USA) as follows: an initial denaturation step (94°C for 10 min) was followed by 30 cycles of amplification (94°C for 1 min, 68°C for 1 min, and 72°C for 1 min) and a final elongation step at 72°C for 10 min. Amplicons were then purified using CleanPCR magnetic beads (Clean NA, GC Biotech B.V., Waddinxveen, the Netherlands) in a 96-well format. The concentration of the purified amplicons was controlled using a NanoDrop spectrophotometer (Thermo Fisher Scientific, Waltham, MA, USA), and a subset of amplicon sizes was controlled on a Fragment Analyzer (AATI, Hialeah, FL, USA) with the reagent kit ADNdb 910 (35–1,500 bp).

Sample multiplexing was performed on the Abridge platform (INRAE, Jouy en Josas, France) by adding tailor-made 6-bp unique indices during the second PCR step at the same time as the second part of the P5/P7 adapters to obtain primer PCR2_P7F and reverse primer PCR2_P7R (**Table 2**). This second PCR step was performed on 50–200 ng of purified amplicons from the first PCR using 2.5 U of a DNA-free Taq DNA polymerase and 1× Taq DNA polymerase buffer. The buffer was completed with 10 nmol of a dNTP mixture (Sigma-Aldrich, St. Louis, MO, USA), 25 nmol of each primer (Eurofins, Luxembourg), and nuclease-free water (Qiagen, Hilden, Germany) up to a final volume of 50 μl. The PCR reaction was carried out on a T100 thermal cycler with an initial denaturation step (94°C for 10 min), 12 cycles of amplification (94°C for 1 min, 65°C for 1 min, and 72°C for 1 min), and a final elongation step at 72°C for 10 min. Amplicons were purified as described for the first PCR reaction. The concentration of the purified amplicons was measured using a NanoDrop spectrophotometer (Thermo Fisher Scientific, Waltham, MA, USA), and the quality of a subset of amplicons (12 samples per sequencing run) was controlled on a Fragment Analyzer (AATI, Hialeah, FL, USA) with the reagent kit ADNdb 910 (35–1,500 bp). Controls were carried out to ensure that the high number of PCR cycles (35 cycles for PCR1 + 12 cycles for PCR2) did not create significant amounts of PCR chimeras or other artifacts. The region of the 16S rDNA gene to be sequenced has a length of 467 bp, for a total amplicon length of 522 bp after PCR1 and of 588 bp after PCR2 (using the 16S rDNA gene of *Escherichia coli* as a reference).

Negative controls were included to assess the technical background using nuclease-free water (Qiagen, Hilden, Germany) in place of the extracted DNA during the library preparation.

All libraries were pooled with equal amounts to generate equivalent numbers of raw reads for each library. The DNA concentration of the pool (no dilution or diluted 10× and 25× in EB + Tween 0.5% buffer) was quantified on a Qubit fluorometer (Thermo Fisher Scientific, Waltham, MA, USA). The pool, at a final concentration between 5 and 20 nM, was used for sequencing.

#### 2.6.3 Sequencing

The pool was denatured (0.1 N NaOH) and diluted to 7 pM. PhiX Control v3 (Illumina, San Diego, CA, USA) was added to the pool at 15% of the final concentration as described in the Illumina procedure. A total of 600 μl of this pool and the PhiX mixture were loaded onto the Illumina MiSeq cartridge according to the manufacturer’s instructions using MiSeq Reagent Kit v3 (2 × 300 bp paired-end reads, 15 Gb output). FastQ files were generated at the end of the run (MiSeq Reporter software, Illumina, San Diego, CA, USA) for quality control. The quality of the run was checked internally using PhiX Control, and then each paired-end sequence was assigned to its sample using the multiplexing index.

#### 2.6.4 Bioinformatic analysis

The resulting sequences were received in the format of de-multiplexed FASTQ file and analyzed using FROG (version 4.0.1) (36). The sequences obtained from the 267 samples were analyzed according to the following workflow: pre-processing (maximum read size, 250; maximum rate of mismatch in the overlap region, 0.1; Vsearch software for merging paired-end reads, 150–450 limits for amplicon sizes; primers, 5′-ACGGRAGGCAGCAG and 3′-AGGATTAGATACCCTGGTA), swarm clustering (distance = 1), chimera removal, and OTU filtering (minimum OTU abundance as proportion or count = 0.00005, as recommended by Bokulich et al., (37), to retain OTUs with at least 0.005% of all sequences; PhiX database for contaminants). Affiliation by blast (version 2.10) with the reference DATABASE SILVA 138.1 (38). The indices Chao1, Shannon, and Simpson were used to determine the alpha diversity. Beta diversity was determined with the weighted UniFrac (39) method and principal coordinate analysis (PCoA). A permutational multivariate ANOVA using distance matrices (vegan::adonis) and Wilcoxon’s test corrected with the Benjamini–Hochberg false discovery rate method were performed to determine whether the paired comparisons of the treatments were significant.

## 3 Results

### 3.1 Larval performance

#### 3.1.1 Survival

**Figure 3:**
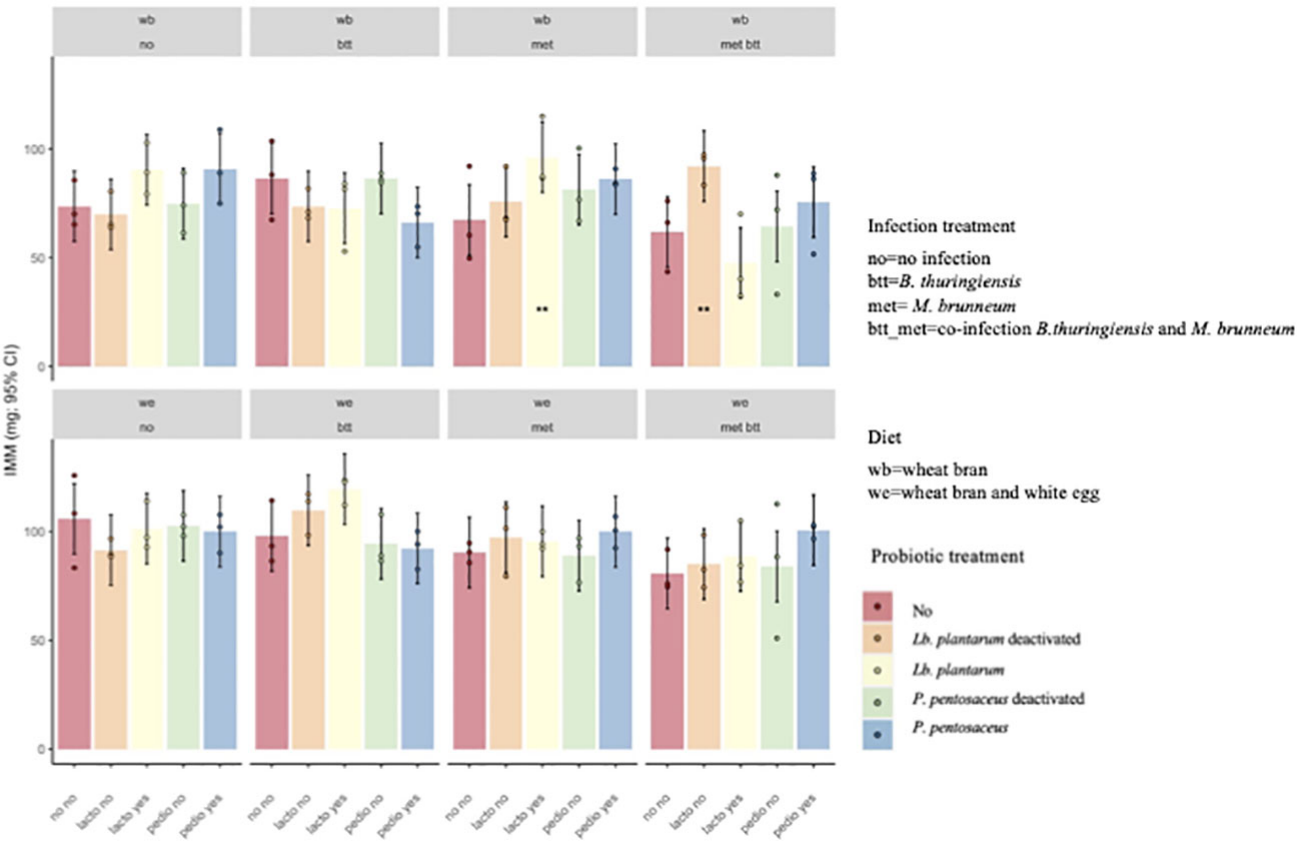
Probability of survival (±95% confidence interval) of *Tenebrio molitor* larvae after 2 weeks of assay. Treatments were the presence in the diet of either live (*Pediococcus pentosaceus* Pp and *Lactiplantibacillus plantarum* Lb) or deactivated probiotic species (*P. pentosaceus* Ppt and *Lb. plantarum* Lbt) on the survival of *T. molitor* larvae fed the wheat bran (*WB*) diet or the wheat bran + egg white (WE) diet and infected with *Bacillus thuringiensis* (*btt*), *Metarhizium brunneum* (*met*), or both pathogens (*met_btt*). Each treatment is the mean of three biological replicates of groups of 20 larvae each. The addition of either live or deactivated *P. pentosaceus* resulted in a significantly higher survival of larvae fed WB in the co-infection conditions (met_btt) (Pp: *p* = 0.0415; Pp_t: *p* = 0.0227). *p ≤ 0.1; **p ≤ 0.05.

Larval survival was recorded on day 14, 11 days after the larvae were moved from the pathogen-inoculated feed, which corresponded to 14 days after contact with the probiotic feed (see **Figure 2**). A model that included the second- and third-order interactions improved the model fit over a model that included only the main effects (*χ* ^2^ = 50.148, *df* = 31, *p* = 0.01619). The results showed significant differences in the survival of *T. molitor* larvae reared on WB with provision of *P. pentosaceus* KVL B19-01, live (Pp) and deactivated (Pp_t), with 92.6% and 94%, respectively, in the co-infection treatment (met_btt) compared with the larvae reared on the WB control diet and exposed to the same two pathogens, where survival was only 74.6% (Pp: *p* = 0.0415; Pp_t: *p* = 0.0227) (**Figure 3**). No effect of the provision of *Lb. plantarum* was found on survival in either infected or uninfected larvae provided with WB or WE (*p* > 0.05). Individuals reared on the WE diet were less susceptible to pathogen infection, as shown by the higher survival probabilities 14 days post-pathogen exposure (**Figure 3**).

#### 3.1.2 Larval weight gain

**Figure 4:**
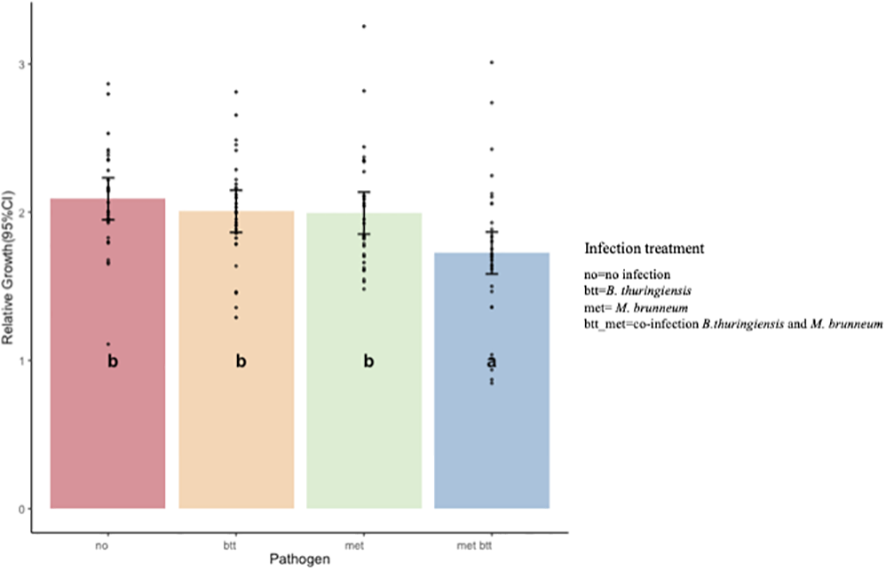
Effect of pathogens on the relative growth of *Tenebrio molitor* larvae reared on all treatments after 2 weeks of assay (14 days). Single infection with either pathogen [*Bacillus thuringiensis* sv*. morrisoni* biovar *tenebrionis* (*btt*) and *Metarhizium brunneum* (*met*)] had no significant impact on relative growth (btt: *p* = 0.84; met: *p* = 0.78) when compared to the control. Co-infection (*met_btt*) resulted in a significantly lower relative growth (*p* = 0.003) than the control. *Bars* show the estimated marginal means and *black dots* the single data points. *Different letters* denote significant differences.

The relative growth (weight gain between days 3 and 14) in the control compared to the other treatments during the assay was significantly influenced by the presence of the pathogens and not by the diet composition or by probiotic addition (ANOVA, type III test: pathogens, *p* = 0.003; diet, *p* = 0.117; probiotic, *p* = 0.137) (**Figure 4**). Significant differences were observed between the met_btt co-infection and the control (*p* = 0.003) and between met_btt and Btt (*p* = 0.033). Similarly, significant differences were observed between the met_btt co-infection and met infection (*p* = 0.045). No significant differences were detected in single infections between the control and Btt infection (*p* = 0.84) or the control and met infection (*p* = 0.78) or between the Btt and met infections (*p* = 0.99).

**Figure 5:**
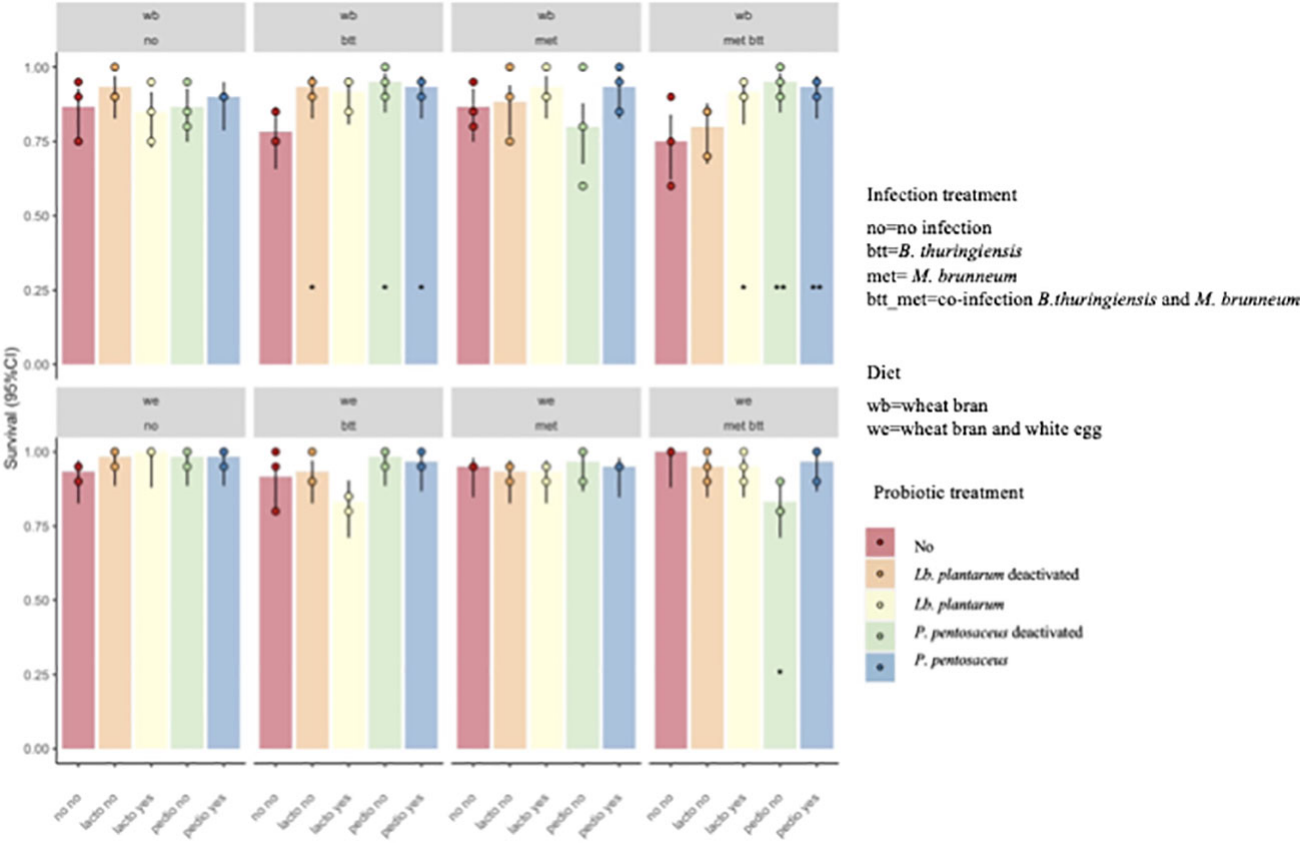
Individual mean mass (IMM) of *Tenebrio molitor* larvae after 2 weeks of growth. *Lactiplantibacillus plantarum* deactivated (*Lb_t*) was the only treatment that had an effect on *Metarhizium brunneum* (*met*) and co-infected larvae (*met_btt*) fed wheat bran (*WB*; *p* = 0.038). The other treatments showed no significant effect on IMM (*p* > 0.05). **p ≤ 0.05.

The IMM was impacted by the interaction of probiotics, diet, and pathogen exposure (*F*_12,80_ = 1.81, *p* = 0.06). Larvae fed the WB diet supplemented with deactivated *Lb. plantarum* (lacto_yes) and infected with *M. brunneum* (met) and those fed the WB diet supplemented with live *Lb. plantarum* (lacto_no) and infected with both pathogens (met_btt) presented higher IMM than the WB with pathogen “control” (*p* = 0.05 and *p* = 0.038, respectively), indicating that the addition of the probiotic reduced the negative impact of the pathogens on larval growth (**Figure 5**).

### 3.2 Microbial community composition

#### 3.2.1 16S rDNA-based bacterial composition of T. molitor larvae fed with probiotics

The effects of probiotic treatment on the composition of the bacterial community were investigated in the two diets (WB and WE) from individuals exposed to different treatments [probiotics and pathogen(s)]. The aim was to identify the level at which the diets and the presence of probiotics and pathogens modify the bacterial composition and the OTU abundance over time after the removal of the treatments. A key interest was also to analyze the extent to which the probiotics or the pathogen could persist in the larvae. The analyses were performed on larvae from day 0 (just after removal from the diet with probiotics), from day 3 (just after removal from the diet with pathogens), and on day 14, corresponding to 11 days fed with WB or WE only. All data were compared with those of the control larvae fed only with WB or WE throughout the whole period from egg to the 14-day assay (**Figures 1**, **2**).

The 16S rDNA Illumina sequencing analysis resulted in a total of 12,890,914 Nb sequences. Pre-processing allowed selecting a number of pre-paired sequences of 1,849,872 Nb (14.35%). After chimera removal (27.5%), the OTU filter processing retained 9,841,546 (92.7%) sequences.

Taxonomy was assigned with the SILVA 138.3 database (38), which determined the affiliation of 100% sequences with 0.005% sequence abundance and an identity of acceptance of 97%. A total of 390 OTUs were obtained from 275 samples and were classified into 6 phyla, 8 classes, 26 orders, 41 families, 85 genera, and 124 species (**Supplementary Table S1**).

On day 0, the microbial composition was mainly represented by *Enterococcus* [Relative abundance (RA%): WE control = 39%, WE_Lp = 6%, WE_Pp = 23%, WE_Lbt = 0%, and WE_Ppt = 6%], *Lactococcus* (%RA: WE control = 22%, WE_Lp = 55%, WE_Pp = 9%, WE_Lbt = 86%, and WE_Ppt = 2%), and *Listeria* (%RA: WE Ppt = 36%) in larvae reared on the WE diet. *T. molitor* fed the WB diet presented prevalence of the genera *Lactococcus* (%RA: WB control = 41%, WB_Lp = 12%, WB_Pp = 14%, WB_Lbt = 7%, and WB_Ppt = 14%) and *Staphylococcus* (%RA: WB control = 53%, WB_Lp = 21%, WB_Pp = 29%, WB_Lbt = 26%, and WB_Ppt = 1%). The family Enterobacteriaceae was also widely present. In all the samples, *P. pentosaceus* was detected from day 0 throughout the assay in all conditions at the species level, while *Lb. plantarum* was not detectable at all.

#### 3.2.2 Impact of diet and probiotics on the microbiota of yellow mealworm

On day 0, the impact of probiotic provision was significantly different for *T. molitor* individuals fed the WB- and WE-based diets. In larvae reared on the WE diet, the alpha indices Chao1 (*p* > 0.05), Shannon (*p* = 0.00056), Simpson (*p* < 0.0001), and InvSimpson (*p* = 0.009) showed significant differences in the species richness of the larvae exposed to the different probiotic treatments. Paired comparisons highlighted differences between the control (no) and *Lb. plantarum* deactivated (Shannon: *p* = 0.008; Simpson: *p* = 0.001; InvSimpson: *p* = 0.016) and in both *P. pentosaceus* live and deactivated (Shannon: *p* = 0.013; Simpson: *p* < 0.0001). In larvae reared on the WB diet, the alpha indices for microbial community composition were not significantly different as a result of probiotic treatment. These findings suggest that the addition of egg white protein (WE diet) had a direct or an indirect influence on the probiotics and the composition of the commensal bacterial microbiota.

**Figure 6:**
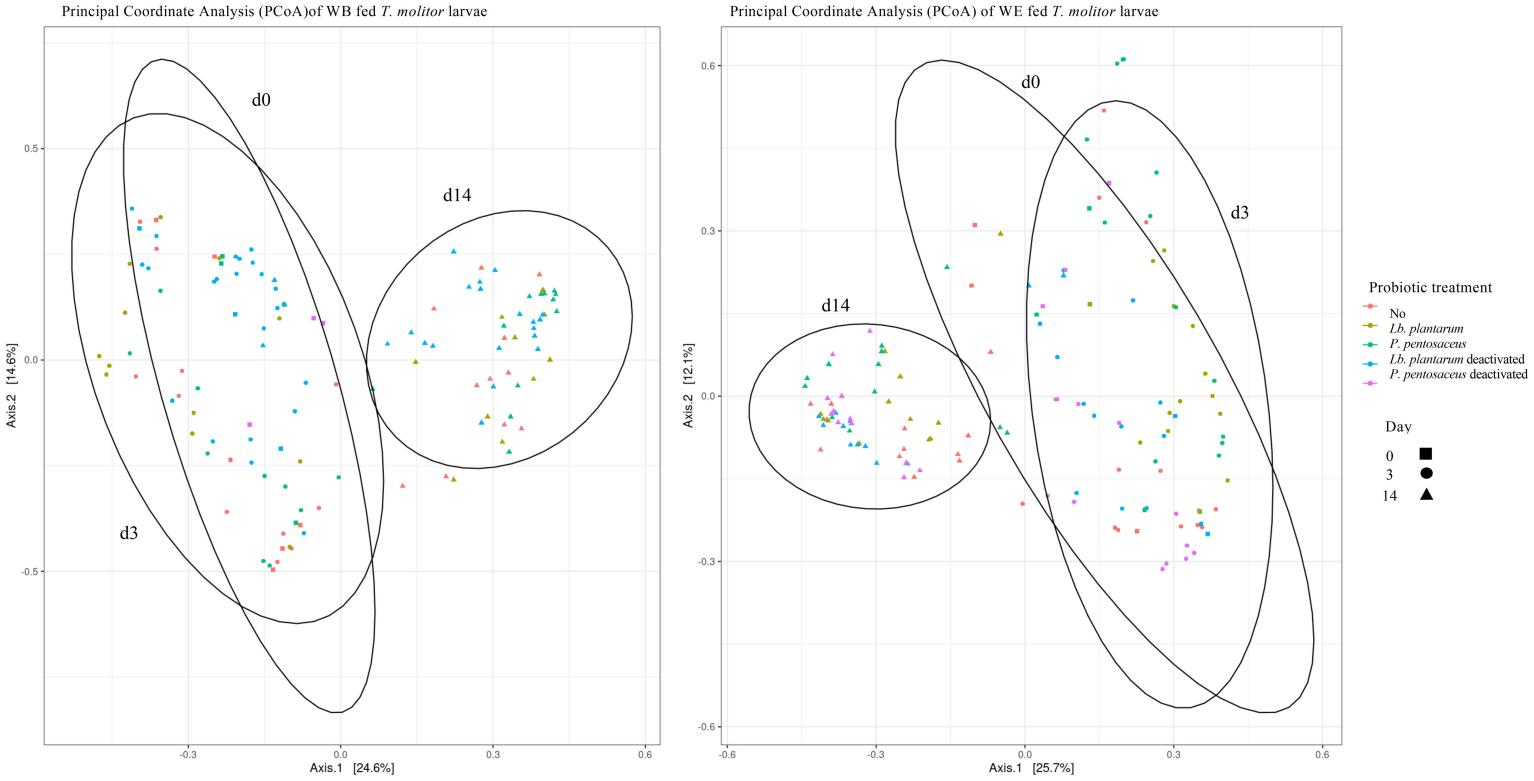
Principal coordinate analysis based on the weighted UniFrac (PCoA) of larvae fed wheat bran (*WB*) (Left) and wheat bran and dried egg white (WE) (Right) given probiotic treatments [control (*red*), *Lactiplantibacillus plantarum* (*yellow*), *Pediococcus pentosaceus* (*green*), *Lb. plantarum* deactivated (*blue*), and *P. pentosaceu*s deactivated (*pink*)] until day 0 (*square*) and then fed the control diet from day 3 (*circle*) and day 14 (*triangle*). Samples present a clustering on day 14, showing differences in the microbial community composition 14 days after treatment removal and in the homogeneity of the microbial community composition between the treatment groups within each day.

Overall, when the microbial communities were compared after 14 days of probiotic removal, the alpha values for OTU composition were not significantly different, suggesting little diversity between the larvae exposed to the different treatments. However, variation was found within each treatment over time. Larvae fed with WE presented significant differences in the alpha values in pairwise comparisons between day 0 and day 14 (Chao1: *p* = 0.002; Shannon: *p* < 0.0001; Simpson: *p* < 0.001) and between day 3 and day 14 (Chao1: *p* < 0.001; Shannon: *p* = 0.0001). Moreover, the WB samples presented significant differences in the alpha values in pairwise comparisons between day 0 and day 14 (Chao1: *p* < 0.0001; Shannon: *p* < 0.0001; Simpson: *p* < 0.001) and between day 3 and day 14 (Chao1: *p* < 0.00001; Shannon: *p* < 0.00001; Simpson: *p* = 0.023). These results are illustrated in the PCoA of the WB and WE diets (**Figure 6**). There is clear clustering of the data. The day 14 clusters were different from those of day 0 and day 3, in which more overlap was observed for both WB and WE. Interestingly, when comparing WB and WE, the clusters were differently positioned in the PCoA.

Comparisons between the live and deactivated probiotic treatments on day 14 showed differences in the relative abundances of the microbial community at the genus level. Larvae fed with live *P. pentosaceus* (WB-Pp) presented higher abundances of species in the genera *Enterococcus* and *Sphingobacterium* compared to those provided with the deactivated strain (WB_Ppt). The same live strain inoculated in the WE diet resulted in higher abundances of *Enterococcus*, *Acinetobacter*, *Enterobacter*, *Erwinia*, and *Pantoea*.

*Lb. plantarum* WJB had no major impact on the microbial community composition in WE-fed larvae compared with the control. On the other hand, in WB supplemented with the live bacteria, the abundance of species in the genera *Sphingobacterium*, *Chryseobacterium*, and *Staphylococcus* was higher, while a lower abundance of *Acinetobacter* was observed.

Comparison between the probiotic treatments and the day of sampling was made using multivariate ANOVA. The two diets showed different results. A significant interaction effect on microbial composition occurred between probiotic treatment and day for larvae fed the WE diet on day 14 (*R* ^2^ = 0.222, *df* = 8, *F* = 2.66, *p* = 0.001). For the WB diet, this interaction was not significant (*R* ^2^ = 0.089, *df* = 6, *F* = 1.5873, *p* = 0.114).

Further analyses were performed on the microbiota composition of the OTUs by applying Wilcoxon’s test corrected with the Benjamini–Hochberg false discovery rate method to compare the probiotic treatments and the day of sampling. Probiotic provision did not result in statistical differences in the OTU composition compared with the control treatments (*p* > 0.05). The microbiota composition of larvae reared on the same probiotic treatments was mainly affected by the time, with differences between day 0 and day 14 being less evident than those between day 3 and day 14 (**Supplementary Figure S2**).

#### 3.2.3 Effects of diet composition on probiotic persistence

The WB- and WE-based diets did not influence the microbial community composition on day 0 in uninfected larvae (multivariate ANOVA: *p* > 0.05). The family Enterobacteriaceae (RA% = 24%) and the genera *Staphylococcus* (%RA = 10%–50%), *Chryseobacterium* and *Sphingobacterium* (%RA = 5%–16%), *Lactococcus* (%RA = 3%–12%), *Pseudomonas* (%RA = 8%), and *Weisella* (%RA = 3%–7%) were the most represented in larvae from all control treatments. Among the diet treatments, a difference in the abundance of the OTUs belonging to the genus *Pediococcus* on day 14 was higher, but not significant, in WE-fed larvae compared to WB-fed larvae (**Supplementary Figure S1**).

#### 3.2.4 Effects of probiotic provision on the microbiota of pathogen-infected larvae

**Figure 7:**
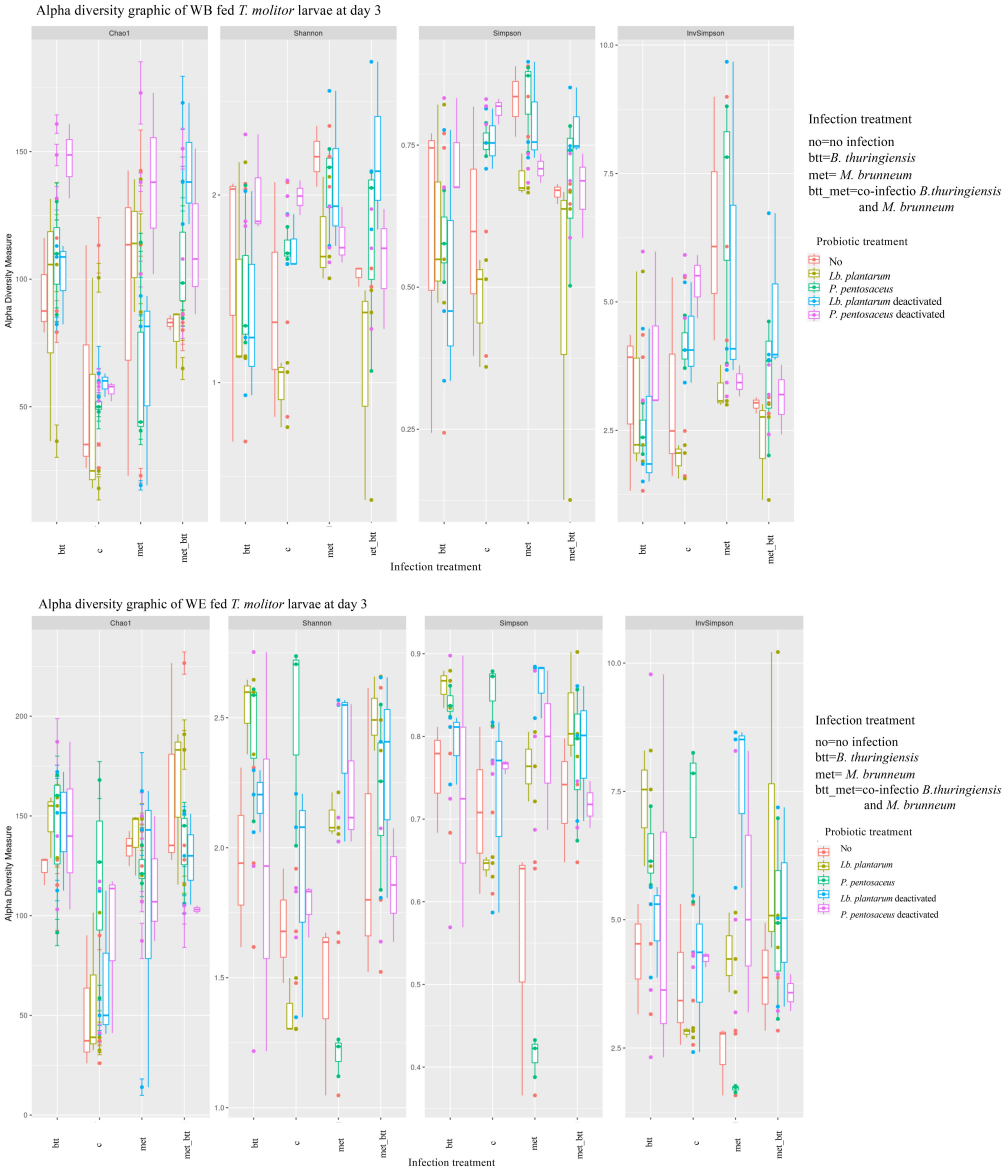
Alpha diversity indices of the microbiota operational taxonomic unit (OTU) data from *Tenebrio molitor* larvae grouped by probiotic treatments [no (control) (*red*), *Lactiplantibacillus plantarum* (*yellow*), *Pediococcus pentosaceus* (*green*), *Lb. plantarum* deactivated (*blue*), and *P. pentosaceus* deactivated (*pink*)] and infections [control, *Bacillus thuringiensis* sv. *morrisoni* biovar *tenebrionis* (*btt*), *Metarhizium brunneum* KVL 12-30 (*met*), and met_btt) on day 3. The box plot shows the OTU abundance between the grouped samples.

*T. molitor* larvae were exposed to pathogens from day 0 to day 3 (72 h), placed in sterile cups, and then provided with non-contaminated feed for 11 days. Overall, the addition of probiotics resulted in significant differences in the larval microbial community abundance on both days 3 and 14 in the case of infection. The probiotic supplementation in WB and WE resulted in significant effects on the microbial community composition on day 3 (*R* ^2^ = 0.2286, *F* = 5.74, *df* = 4, *p* = 0.0001; *R* ^2^ = 0.2394, *F* = 6.7579, *df* = 4, *p* = 0.0001) and on day 14 (*R* ^2 ^= 0.1735, *F* = 4.0275, *df* = 4, *p* < 0.00001; *R* ^2^ = 0.1673, *F* = 4.831, *df* = 4, *p* < 0.00001). When considering the effect of probiotic species on pathogen-infected larvae in the two diet treatments on day 3, the analysis showed no significant effects in WB-fed larvae (*p* > 0.05), but significant differences in WE-reared larvae (*R* ^2^ = 0.0739, *F* = 2.9021, *df* = 12, *p* = 0.0001). Indeed, the microbial community of larvae exposed to single infection with either Btt or met or to co-infection presented different microbial community compositions on day 3 compared with uninfected individuals in WE (Chao1: control-Btt, *p* < 0.0001; control-Btt/met, *p* = 5.468e−7; control-met, *p* = 0.00031). In WB diet treatments, the effect of pathogens on the microbiota composition was less pronounced than that in WE, as examined based on the *p*-values (Chao1: control-Btt, *p* = 0.00190; control-Btt/met, *p* = 0.02439; Shannon: control-met, *p* = 0.02544; InvSimpson: control-met, *p* = 0.01369) (**Figure 7**).

After 3 days, in all the control larvae exposed to *M. brunneum*, *Lactococcus* (%RA = 46%) was the most abundant genus, followed by *Weissella* and *Staphylococcus* and the family Enterobacteriaceae. The controls presented similar microbial composition, with the *Chryseobacterium* and *Sphingobacterium* genera detected with RA ranging from 20% to 53% in infected larvae on day 14. Individuals reared on probiotic treatments presented lower RA of the same genus (%RA < 10%). *Bacillus* species had RA of 3% in infected individuals on day 3, with lowered values in all probiotic-treated larvae (<2%) and were no longer detected on day 14. *Bacillus* species were completely absent in samples fed the WE or WB diet supplemented with deactivated probiotic species.

Interactions between probiotic treatments and infections were evident at the end of the experiment (*R* ^2^ = 0.3478, *F* = 3.3468, *df* = 12, *p* < 0.0001; *R* ^2^ = 0.2941, *F* = 2.4474, *df* = 12, *p* < 0.0001). Indeed, the effects of the treatments on OTU abundance were still detectable in the control larvae and in those infected with Btt (Chao1: *p* = 3.978E−06; Shannon: *p* = 1.446E−06; Simpson: *p* = 0.002475; InvSimpson: *p* < 0.0001). On day 14, WE-fed larvae infected with Btt, met, or with both pathogens presented significant differences in microbial community composition (met-Btt/met: Chao1, *p* < 0.0001; Shannon, *p* = 0.00997; met-Btt: Chao1, *p* = 0.00814; Shannon, *p* = 0.00577; InvSimpson, *p* = 0.00094).

The same trend was not observed in WB-fed larvae, for which the infection treatments did not show any significant impact on the microbial composition on day 14 (*p* > 0.05).

## 4 Discussion

The main objectives of the study were, firstly, to investigate whether the addition of probiotics to the feed in the early development stages of *T. molitor* would protect the larvae from any harmful impact of the entomopathogens; secondly, to analyze whether a protein-enriched diet could similarly increase larval health and performance; and, lastly, to obtain information about the impact of the treatments on the larval microbiota composition and the persistence of probiotics and entomopathogens after the exposure. The experimental setup consisted of 10 different treatments, five based on WB and five on WB with the addition of egg white. Each was supplemented with the potential probiotic bacteria *P. pentosaceus* KVL B19-01 or *Lb. plantarum* WJB in freeze-dried form, either live or deactivated. The effects of the probiotic strains on the larvae of the yellow mealworm were studied by exposing the larvae to one of two entomopathogens or to a combination of both. The recorded data included larval survival, larval growth (weight and IMM), and impact on the bacterial microbiota composition.

Firstly, with respect to the potential probiotic effects on the survival of pathogen-exposed larvae, our study showed only a minor positive impact, which might partly be due to the fact that both pathogens showed low (for Btt) or no pathogenicity for *M. brunneum*, on the examined 20-mg larvae. Indeed, Btt infection in control larvae without probiotics resulted in 75% and 85% survival in the WB and WE treatments, respectively. On the other hand, an increased larval survival probability was recorded in the case of Btt alone and in co-infection when fed with both live and deactivated *P. pentosaceus* KVL B19-01 in the WB diet, while no positive effect was found in the WE diet; a weak negative impact was recorded with deactivated *P. pentosaceus.* The provision of *Lb. plantarum* WJB resulted in a small positive impact on larval survival in WB treatments with Btt or co-infections. Overall, the infected WE-fed individuals showed high survival (>75%). The effect of the inclusion of dried egg white in the diet on larval survival suggests a stimulatory effect of this protein source on the insect immune system. The importance of proteins in insect feed, insect development, and behavior has been explored in several species (40). In this case, the presence of lysozyme among the proteins of the egg white (41) may have had an antimicrobial effect on the larvae (42).

The relative growth of the larvae was mainly affected by the presence of pathogens and not by the probiotic treatments or the diet composition. The presence of both pathogens resulted in the lowest weight gain, followed by single infection with Btt or *M. brunneum*. Interactions between pathogens and the Btt toxin crystal and the insect gut could activate energy-consuming immune and metabolic pathways (43). Similarly, the activity of Cry toxins has been known to induce reduced feed uptake, resulting in decreased weight gain and lower general fitness (44).

A positive impact of *Lb. plantarum* WJB was recorded since an increased developmental rate, expressed as the IMM, was found in infected larvae fed this probiotic compared to the other infection treatments.

The methodology differed in the present study, and our results appeared not in accordance with those of previous research that found both live and deactivated *P. pentosaceus* increasing the larval weight of *T. molitor* (25). However, live *P. pentosaceus* increased larval survival following challenge by a pathogen, as observed by Dahal et al. (45). The mechanisms behind the observed results are not yet fully understood, especially in the case of the deactivated *P. pentosaceus* cells. However, other studies have demonstrated that, in some cases, the addition of dead microbes can be as effective as the addition of live ones by serving as a source of protein or by acting as an immune stimulator (46, 47).

With regard to the gut microbial composition of the larvae, the results showed that *Lb. plantarum* was not detectable in the insect. This effect is similar to that observed by Storelli et al. (27) in a study on *Drosophila melanogaster*, where the provided bacterial species was not able to persist in the gut lumen after its removal from the feed. On the other hand, *P. pentosaceus* was detectable during the whole experiment in all samples from all treatments, indicating its natural presence in the microbiota community of *T. molitor*. Both the live and deactivated forms of *P. pentosaceus* and *Lb. plantarum* had an impact on the abundance of the microbiota community of the larvae. After probiotic treatment removal and rearing on the control diets, the bacterial species composition of the microbiota and their abundance were comparable to those in previous studies in which the main phyla were represented by Proteobacteria, Firmicutes, and Bacteroides (26, 48).

The presence of lactic acid bacteria, including species of the genera *Lactococcus*, *Lactobacillus*, and *Weissella*, could increase the presence of antimicrobial molecules in the gut, such as bacteriocins, organic acids, hydrogen peroxide, acetaldehyde, acetoin, and carbon dioxide (49). These may confer protection to the yellow mealworm from entomopathogen infections, as observed in honeybees and *Galleria mellonella* (50–52). Interestingly, larvae exposed to fungal infection with *M. brunneum* resulted in higher abundance of *Weisella* and *Lactococcus* species, suggesting a protective role due to their gut colonization ability (53) and the production of organic acids and nisin (49, 54, 55) with proven antifungal activities, which might explain the low pathogenicity of *M. brunneum* observed in this study.

The presence of *Acinetobacter* and *Sphingobacterium* species might be explained by the preference of *T. molitor* for cereal-based diets, the digestion of which requires the action of cellulolytic enzymes, provided as in other insect species by gut symbionts such as the European corn borer (*Ostrinia nubilalis*) and the Colorado potato beetle (*Leptinotarsa decemlineata*) midguts (56).

The fact that *P. pentosaceus* was found in the larvae from all treatments may also explain why its addition in the feed did not result in increased performance. Further studies are needed to compare the strains at the genome level. Indeed, the *P. pentosaceus* of the *T. molitor* strain used in this study has not been isolated yet. It is worth mentioning that some *P. pentosaceus* strains are already permitted as animal feed additives, such as the NCIMB 30168 strain from Volac International Ltd. (57). It would be of interest to analyze whether the presence of *P. pentosaceus* in *T. molitor*-based poultry or fish feed would result in increased performance. In addition, exploring the potential probiotic effect of other bacterial species from the gut, such as Clostridiaceae and the Enterococcaceae and Enterobacteriaceae families, could be considered due to their abundance in the microbiota of yellow mealworm and their proven probiotic effect on some species in coleopterans such as *T. molitor* (26) and *Tribolium castaneum* (58), as well as in insects from other orders such as *Musca domestica* L. (Diptera: Muscidae) (59). However, specific precautions would be required prior to approval as probiotics since important pathogens belong to the same families (60, 61).

Gut microbiota–host interactions are important for increasing digestion and nutrient absorption (62). As has been observed in previous studies, genus abundance also depends on the diet composition provided. Indeed, in larvae fed a higher dietary protein content, the presence of species able to produce cellulolytic enzymes, such as *Acinetobacter* spp., was reduced (63). In our study, larvae fed the WB diet showed differences in microbial OTU abundance, resulting in higher loads of *Pediococcus* spp. on day 0 compared to larvae fed WB with dried egg white (WE diet). If *Pediococcus* is needed for good digestion of WB, this might explain the addition of egg white protein not increasing the larval growth rate in the present study.

Furthermore, an increased larval performance may not be directly due to the probiotic acting as a nutrient source; rather, the mechanism could be the direct interaction of *P. pentosaceus* KVL B19-01 with larval immunological or physiological mechanisms, leading to increased growth and immune resistance to pathogens (64). The ability of *P. pentosaceus* to produce and excrete antimicrobial secondary metabolites such as pediocins (65, 66) might play an important role in shaping the insect microbiota dynamics by favoring probiotic gut colonization and avoiding pathogen proliferation (45). The mechanisms related to pediocin production in the gut environment of the yellow mealworm still need to be investigated, taking into consideration the gut pH (67) and the mass-rearing environmental conditions that might affect the insect microbiota dynamics (68). Further studies should also consider the impact of the probiotics on the whole life cycle of *T. molitor*, as earlier reported (25, 45). Moreover, the study of the persistence of pathogens and probiotics in the larvae was possible due to the controlled experimental conditions, characterized by the timed provision of feed and removal of frass (**Figure 2**). In real-life insect mass rearing, it is more complicated to measure and control the conditions; therefore, the application of GMP to ensure insect and human safety (14) is needed. Similarly, the possibility to monitor microbial species in order to assess the quality of the rearing system along with timed probiotic provision would help in maintaining healthy production conditions (18, 34).

## 5 Conclusions

The use of agricultural by-products as insect feed substrates might increase the risk of entomopathogen infections in the mass rearing of *T. molitor*. In this study, we demonstrated that, by supplementing the feed from egg hatching to the 20-mg stage with live and deactivated forms of an isolate of the bacterium *P. pentosaceus*, it is possible to reduce the lethal impact of fungal and bacterial pathogens particularly in co-infection conditions. However, the same effect was not observed with *Lb. plantarum*, although it improved larval mass gain, indicating specificity in host–symbiont relationships. Moreover, *T. molitor* larvae did not show improved weight gain after probiotic provision in non-infection conditions, possibly due to the fact that the provided *Lb. plantarum* did not establish or because the high nutritional value of the WB diet overruled the beneficial effect reported in previous studies in undernourished insects. In addition, the dynamics of the microbiota composition indicated the inability of both the added pathogens and probiotics to persist or increase in abundance in the larvae after their provision ended, indicating the importance of the microbial community homeostasis in insect health. Overall, probiotic bacteria could be applied in insect mass-rearing facilities as a preventive treatment to reduce the risk of entomopathogen infection.

## Data availability statement

The sequencing data are available on NCBI SRA database http://www.ncbi.nlm.nih.gov/bioproject/1059781 and the original contributions presented in the study are included in the article/**Supplementary Material**. Further inquiries can be directed to the corresponding author.

## Ethics statement

The manuscript presents research on animals that do not require ethical approval for their study.

## Author contributions

CS: Conceptualization, Data curation, Investigation, Project administration, Visualization, Writing – original draft, Writing – review & editing. PH: Conceptualization, Writing – review & editing. AL: Writing – review & editing. ARe: Investigation, Writing – review & editing. WB: Data curation, Writing – review & editing. ARi: Data curation, Writing – review & editing. AB-J: Funding acquisition, Resources, Writing – review & editing. JvL: Funding acquisition, Resources, Writing – review & editing. CN-L: Conceptualization, Funding acquisition, Project administration, Resources, Writing – review & editing.
