# Supplementary material for: Minor impact of probiotic bacteria and egg white on Tenebrio molitor growth, microbial composition, and pathogen infection
Source: Front Insect Sci. 2024 Mar 1;4:1334526. doi: 10.3389/finsc.2024.1334526 (PMC10926391; doi:10.3389/finsc.2024.1334526)
Supplement: Supplementary file 1 [file DataSheet_1.pdf]

Supplementary Material

Minor impact of probiotic bacteria and egg white on *Tenebrio molitor* growth, microbial composition and pathogen infection

Carlotta Savio[1,2,6*], Pascal Herren[3,4,6], Agnès Rejasse[1], Alfredo Rios[5], William Bourelle[1], Annette Bruun-Jensen[6], Antoine Lecocq[6], Joop J.A. van Loon[2], Christina Nielsen-LeRoux[1]

*** Correspondence:** carlottasavio2@gmail.com

1 Figures

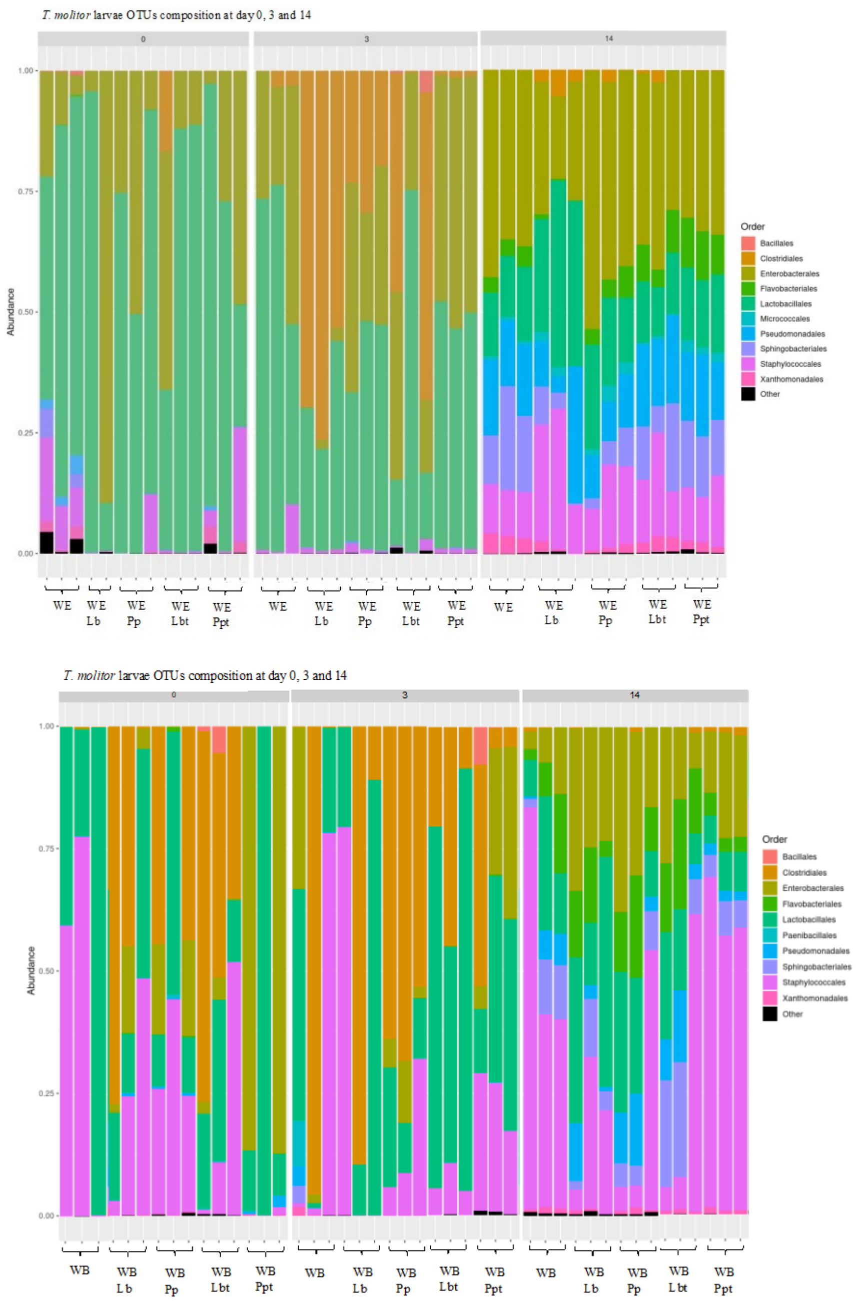

Supplementary Figure 1. OTUs relative abundances representing the main baterial orders found in *T. molitor* larvae reared on wheat bran (WB) or wheat bran and egg white (WE) diet supplemented with *P. pentosaceus* or *Lb. plantarum* live (Lb, Pp) or deactivated (Lbt, Ppt) forms. The sampling was performed when the larvae reached 20 mg weight (day 0) and at 3 and 14 days after they were moved to WB or WE without probiotics, for each condition analyses are shown from three individual larval extractions.

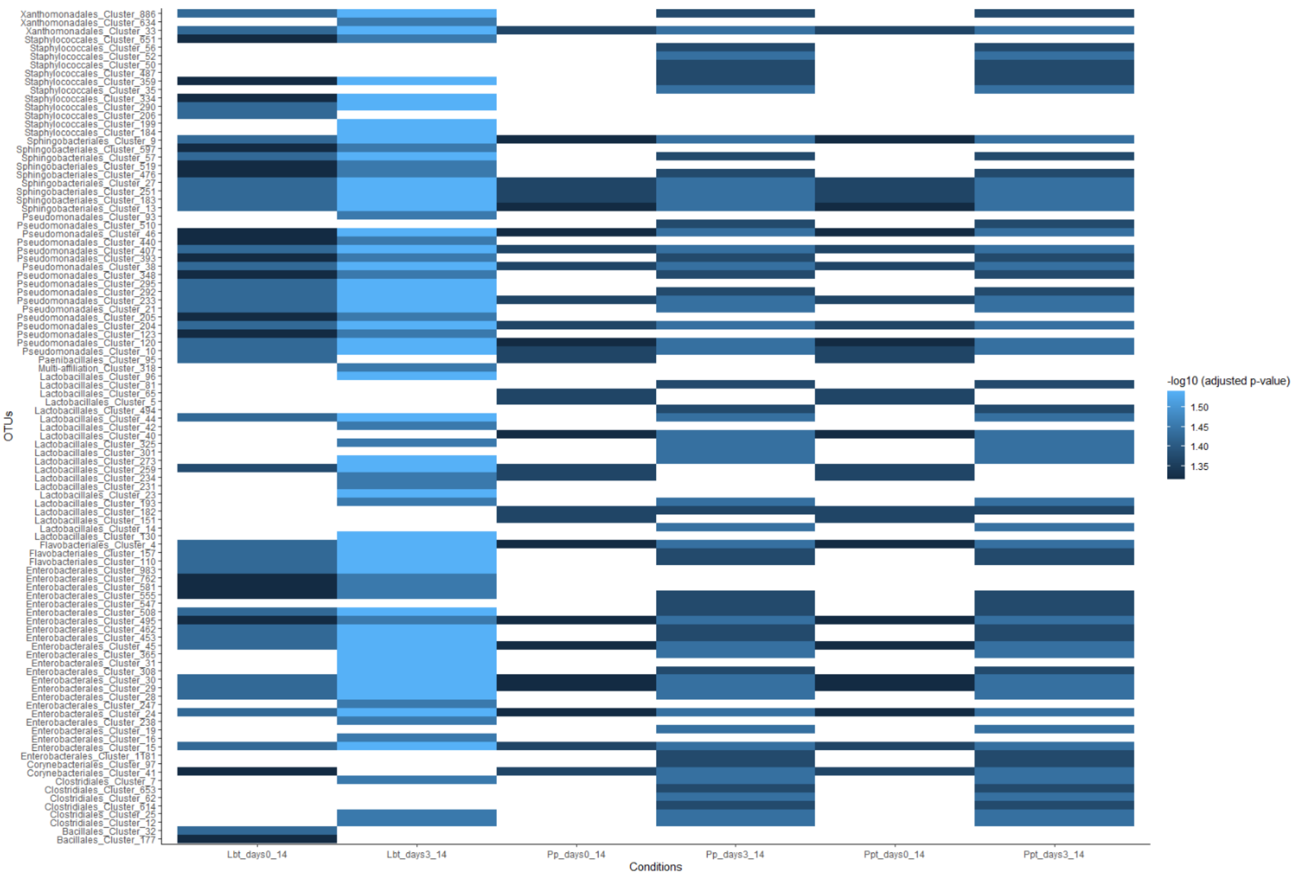

Supplementary Figure 2. Heat map showing OTUs abundances' differences in *T. molitor* larvae fed with wheat bran (WB) or wheat bran and egg white (WE) and treated with *P. pentosaceus* or *Lb. plantarum* in live (Lb, Pp) or deactivated (Lbt, Ppt) forms. All the data were analyzed with Wilcoxon tests corrected with the Benjamini-Hochberg false discovery rate correction method to understand the influence of the probiotic treatment and the time after the last ingestion of probiotics. The heatmap exhibits only the treatments where significative differences in some OTUs' abundance were found ($P < 0.05$). The blue gradient shows for which clusters the adjusted P value was lower than 0.05 while the blank squares indicate that the P value found for these clusters was higher than 0.05, meaning that there is no significative difference in abundance for these OTUs between the two conditions tested. The probiotics did not have a significative impact on OTU's abundance. The data represented in this figure show an influence of the day after the last probiotic ingestion on several OTU's abundance. Main differences were found both between day 0 and day 14 and between day 3 and day 14.
